# Nightmare disorder and REM sleep behavior disorder in inflammatory arthritis: Possibility beyond neurodegeneration

**DOI:** 10.1002/brb3.1230

**Published:** 2019-02-15

**Authors:** Luca Baldelli, Olga Addimanda, Marco Burattini, Giacomo Chiaro, Veronica Brusi, Elettra Pignotti, Riccardo Meliconi, Federica Provini

**Affiliations:** ^1^ Department of Biomedical and NeuroMotor Sciences (DiBiNeM) University of Bologna Bologna Italy; ^2^ Medicine & Rheumatology Unit IRCCS Rizzoli Ortopaedic Institute Bologna Italy; ^3^ Neurological Clinic Marche Polytechnic University Ancona Italy; ^4^ Sleep and Epilepsy Center Neurocenter of Southern Switzerland, Civic Hospital of Lugano Lugano Switzerland; ^5^ IRCCS Istituto delle Scienze Neurologiche di Bologna Bologna Italy

**Keywords:** Inflammatory arthritis, Neurodegeneration, Nightmares, Prevalence, RBD, Sleep

## Abstract

**Objectives:**

To investigate the prevalence of REM sleep behavior disorder (RBD) in patients with inflammatory arthritis (IA) to ascertain if RBD could be an internal red flag signaling a fluctuating state of inflammation based on the theory of “protoconsciousness”.

**Materials & Methods:**

One hundred and three patients with a confirmed diagnosis of IA were consecutively recruited. The patients underwent general (IA activity, functional status, laboratory tests) and neurological evaluations. A neurologist investigated RBD and REM sleep parasomnias in a semi‐structured interview. Sleep quality was assessed with the Pittsburgh Sleep Quality Index, while the risk of obstructive sleep apnea syndrome (OSAS) was evaluated with the Berlin questionnaire. Beck Depression Inventory II and State‐Trait Anxiety Inventory investigated depression and anxiety.

**Results:**

Patients had a mean age of 53.7 ± 14.6 years, 65% were women; 57.3% were in a clinically active phase of IA. Two women fulfilled ICSD‐3 criteria for RBD appearing 11 years after and 20 years before IA onset respectively. 31 patients scored positive for nightmare disorder (ND), 8 for recurrent isolated sleep paralysis. 65 (63.1%) patients reported poor sleep quality and 25 (24.3%) resulted at high risk for OSAS. 32 (31.0%) patients scored positively for depression or anxiety.

**Conclusions:**

The prevalence of RBD in patients with IA did not differ from that in the general population, whereas ND presented a 2‐fold increased prevalence. Whether RBD can be considered a red flag signaling an internal danger remains an open question, while ND may be a new player in this intriguing relation.

## 1 INTRODUCTION

REM sleep behavior disorder (RBD) is a REM sleep parasomnia characterized by loss of physiological muscle atonia during REM sleep and dream enacting behavior (AASM, 2014). REM sleep behavior disorder (RBD) has an estimated prevalence of 0.5% in the general population (Ohayon, Caulet, & Priest, 1997) and 2% in older adults, predominating in males (AASM, 2014), although recent evidence reported only a 52.4% male prevalence in the general population ( Haba‐Rubio et al., 2018).

Longitudinal observation of RBD patients showed that up to 81% of them developed neurodegenerative diseases, in particular α‐synucleinopathies, in 16 years (Schenck, Boeve, & Mahowald, 2013). The prevalence and characteristics of RBD have never been investigated outside a neurological setting.

The pathogenesis of RBD is complex and still not fully clarified. Animal and lesional studies demonstrated how loss of physiological muscle atonia and retention of muscle tone in REM sleep (REM sleep without atonia – RSWA) associated with the disinhibition of mesencephalic motor pattern generators resulting in the subsequent release of the dream enacting behaviors are necessary conditions for RBD to develop (Luppi, Clement, Valencia Garcia, Brischoux, & Fort, 2013). In addition, abnormal dream mentation may stem from a concomitant limbic system dysfunction, particularly in the amygdala (Cornelius et al., 2011). Dreaming processes and REM sleep, interconnected constituents of physiological sleep, could signal a deeper dysfunction at neuronal and circuital level (Luppi et al., 2013).

REM sleep has been considered a constituent of “protoconsciousness” (Hobson, 2009). Through the creation of a virtual representation of reality based on experience on the “outside world” (dreams), REM sleep could anticipate events and perceptions yet to occur and hence prepare the individual to adopt the most appropriate behaviors during wakefulness, especially in situations of danger (Hobson, 2009; Horowski, Benes, & Fuxe, 2004). In line with this hypothesis, a REM sleep disorder such as RBD could represent an internal red flag signaling a condition with a negative impact exposing us to constant or prominent danger.

Autoimmune inflammatory diseases include autoimmune inflammatory arthritis (IA), a non‐neurological but invalidating chronic inflammatory disease such as rheumatoid arthritis (RA) and seronegative spondyloarthropathies (SpA) (namely ankylosing spondylitis ‐ AS and psoriatic arthritis ‐ PsA). These conditions exemplify both a constant and prominent danger due to a chronic state of inflammation characterized by periodic relapses. Little evidence is available on sleep in autoimmune inflammatory arthritis (IA). Few studies have investigated the impact of nocturnal sleep quality on quality of life (Batmaz et al., 2013; Westhovens, Elst, Matthys, Tran, & Gilloteau, 2014), while none have specifically searched for RBD in this population. Therefore, the main aim of this study was to investigate the prevalence of RBD in patients with IA.

## 2 METHODS

### 2.1 Study population

From April 2014 to November 2016 we consecutively recruited 103 patients with a diagnosis of IA attending the outpatient clinic of the Medicine and Rheumatology Unit, DIBINEM, Istituto Ortopedico Rizzoli, University of Bologna.

Inclusion criteria were a confirmed diagnosis of RA (Aletaha et al., 2010) or PsA or AS (Rudwaleit et al., 2009, 2011) for at least two years and age between 20 and 75 years. Exclusion criteria were a history of alcohol or drug abuse/dependence, chronic use of antidepressants at the time of the visit and documented neurological diseases (e.g., stroke, neurodegenerative diseases, epilepsy, demyelinating diseases, etc.).

### 2.2 Ethical approval and patient consent

All patients enrolled in this study agreed to participate by signing the informed consent form. The Ethical Committee of Istituto Ortopedico Rizzoli agreed to the project (protocol n. 0014451 –17/04/2014). This study was performed in accordance with the Declaration of Helsinki.

### 2.3 Procedures

Each recruited patient received a general clinical evaluation including complete medical history‐taking and neurological examination performed by a trained neurologist to exclude any signs suggestive of neurological diseases (e.g. subtle parkinsonism).

The ongoing IA was studied assessing clinical disease activity using the Disease Activity Score 28 (DAS28) (Van derHeijde, Van't Hof, VanRiel, & Van dePutte, 1993) for RA and PA, Bath Ankylosing Spondylitis Disease Activity Index (BASDAI) (Garrett et al., 1994) for AS, discriminating between active and inactive disease (Garrett et al., 1994). Patients’ functional status (disability) was assessed using the Health Assessment Questionnaire (HAQ). Laboratory evaluations including complete blood count (CBC), inflammation reactants (C‐reactive protein – CRP and erythrocyte sedimentation rate – ESR), liver (aspartate aminotransferase – AST and alanine aminotransferase – ALT) and renal (serum creatinine) function tests were performed.

A neurologist investigated clinical RBD and other REM sleep parasomnias based on an interview following the criteria of the International Classification of Sleep Disorders, Third edition (ICSD‐3). Patients who fulfilled the 4 criteria for clinical RBD (probable RBD – pRBD) underwent a semi‐structured questionnaire for RBD (Scaglione et al., 2005) to examine the semeiological and temporal features of RBD episodes. In particular, we investigated the temporal relationship between RBD onset and IA activity.

Moreover each patient completed the Pittsburgh Sleep Quality Index (PSQI) (Curcio et al., 2013) for a global assessment of sleep quality and the Berlin questionnaire (Netzer, Stoohs, Netzer, Clark, & Strohl, 1999) to quantify the risk of obstructive sleep apnea syndrome (OSAS). Affective disorders (depression and anxiety) were evaluated with the Beck Depression Inventory II (BDI‐II) (Beck, Steer, Ball, & Ranieri, 1996) and State‐Trait Anxiety Inventory (STAI) (Spielberger, Vagg, Barker, Donahan, & Westberry, 1980).

### 2.4 Database and statistical analysis

A designated database was created for the study collecting demographic data, clinically relevant comorbidities, laboratory data and questionnaire results. All continuous normally distributed data were expressed in terms of the mean and standard deviation of the mean, while not normally distributed data were expressed in terms of the median and quartiles; the categorical data were expressed as frequency and percentages.

Descriptive analysis was performed on these data and the main features of the individual RBD events were described. The results were further compared with epidemiological evidence in the literature for both RBD prevalence in the general population and sleep features in IA.

The Kolmogorov Smirnov test was performed to test the normality of continuous variables. The Levene Test was performed to test homoscedasticity. The ANOVA test was performed to assess the between groups differences of continuous, normally distributed and homoscedastic data, the Mann Whitney test was used otherwise. Fisher's chi square test was used to investigate the relationships between dichotomous variables. Pearson's chi square test evaluated by Monte Carlo Methods for small samples was performed to investigate the relationships between grouping variables. For all tests *p* < 0.05 was considered significant. All statistical analyses were performed using SPSS v.19.0 (IBM Corp., Armonk, NY, USA).

## 3 RESULTS

### 3.1 Clinical and demographic findings

The sample comprised 103 patients (36 men and 67 women), mean age: 53.7 ± 14.6 years (range 21–75). 64 of them were diagnosed with RA and 39 with SpA (26 PA and 13 AS). Comparing gender distribution in the two diagnostic groups, males were significantly predominant in SpA (RA vs. SpA: 20.3% vs. 59.0%, *p* < 0.0005) (Table 1). SpA patients were significantly younger (*p* = 0.028) and had a higher body mass index (BMI) (*p* = 0.032) in comparison to RA patients.

**Table 1:** Demographic and clinical characteristics of the study cohort stratified by IA type

	Total (103 pts)	RA (64 pts)	SpA (39 pts)	*p*
Demographic Variables				
Age (years; mean ± *SD*)	53.7 ± 14.6	56.2 ± 14.0	49.7 ± 14.8	**0.028**
Men (%)	36 (35.0)	13 (20.3)	23 (59.0)	**<0.0005**
BMI (Kg/m^2^; mean ± *SD*)	24.7 ± 4.4	24.0 ± 3.8	25.9 ± 5.1	**0.032**
Alcohol consumers^a^ (%)	35 (34.0)	23 (35.9)	12 (30.8)	0.671
Smokers/Early Former Smokers^b^ (%)	23 (22.3)	15 (23.4)	8 (20.5)	0.811
IA Variables				
IA onset (years; mean ± *SD*)	43.4 ± 15.6	42.0 ± 18.6	38.9 ± 17.6	0.233
IA duration (years; mean ± *SD*)	10.5 ± 9.0	11.4 ± 9.7	9.2 ± 7.6	0.412^c^
IA in active phase (%)	59 (57.3)	38 (59.4)	21 (53.8)	0.68
Comorbidities				
Hypertension (%)	26 (25.2)	16 (25.0)	10 (25.6)	>0.999
Diabetes (%)	8 (7.8)	4 (6.3)	4 (10.3)	0.707
IA Treatment				
Conventional DMARD (%)	60 (58.3)	42 (65.6)	18 (46.2)	0.062
Biologic DMARD (%)	46 (44.7)	25 (39.1)	21 (53.8)	0.158
Glucocorticoids (%)	31 (30.1)	24 (37.5)	7 (17.9)	**0.045**
Concurrent Medications				
Benzodiazepines (%)	3 (2.9)	3 (4.7)	0 (0)	0.287
Analgesic (%)	15 (14.6)	12 (18.8)	3 (7.7)	0.156
Antihypertensive (%)	9 (8.7)	6 (9.4)	3 (7.7)	>0.999

Note

RA: Rheumatoid Arthritis; SpA: Seronegative Spondyloarthropathies; *SD*: Standard Deviation; BMI: Body Mass Index; IA: Inflammatory Arthritis; DMARD: Disease‐Modifying AntiRheumatic Drug.

Bold values are used to identify statistically significant results (*p*<0.05).

a Patients who answered “yes” to the following question: “Do you have a daily intake of alcohol (e.g. a glass of wine at dinner)?”

b Patients either current smokers or who quit smoking less than 10 years ago.

c The Mann‐Whitney test was used for this variable.

At the study evaluation, 59 (57.3%) patients presented with a clinically active phase of IA, without any difference between the RA and SpA groups. Mean DAS‐28 was 3.10 ± 1.46 in RA patients and 2.60 ± 1.51 in PsA patients; BASDAI scored 3.08 ± 2.56 in AS.

Laboratory values showed slightly increased reactants of inflammation: CRP (4.0, 1.0–10.0 mg/L; median, first quartile‐third quartile) and ESR (15.0, 8.0–25.0 mm/hr), which were significantly increased in patients in an active phase of IA (CRP active vs. inactive: 4.0, 2.0–13.0 mg/L vs. 3.0, 1.0–7.0 mg/L, *p* = 0.049 – ESR active vs. inactive: 21.0, 13.5–36.5 mm/hr vs. 9.0, 4.0–13.0 mm/hr, *p* < 0.005). Patients also had overall preserved liver (AST: 22.2 ± 14.7 U/L, ALT: 24.2 ± 22.3 U/L) and renal (Creatinine: 0.82 ± 0.31 mg/dl) functions. CRP was the only value to show a significant difference between RA and SpA (RA vs. SpA: 2.8, 0.5–5.2 mg/L vs. 6.5, 2.0–20.5 mg/L, *p* = 0.006).

Eighty‐eight patients were under specific treatment for IA: 42 were in therapy with only conventional disease‐modifying antirheumatic drugs (DMARDs), 28 used only biological DMARDs, 18 patients used both, without differences between RA and SpA groups. General anti‐inflammatory therapy for comorbidities showed a more frequent usage of steroids in RA patients (*p* = 0.045). Clinical and demographic features are summarized in Table 1.

### 3.2 Affective disorders questionnaires

Twenty‐three (22.3%) patients scored positive for depression at BDI‐II, 6 of them reported moderate scores and two severe. Four patients had a history of depression, all of them scored positive at BDI‐II. STAI was positive in 11 (10.7%) patients for state anxiety; trait anxiety was present in 7 (6.8%) patients (Table 2). To sum up, 32 (31.0%) patients scored positively for at least one affective disorder. There were no significant differences for these variables between the RA and SpA subgroups.

**Table 2:** Results of affective disorders questionnaires further stratified by IA type

	Total (103 pts)	RA (64 pts)	SpA (39 pts)	*p*
Depression – BDI‐II				
Positive (%)	23 (22.3)	14 (21.9)	9 (23.1)	>0.99
Score (mean ±SD)	8.7 ± 7.2	8.4 ± 7.6	8.6 ± 6.6	0.842
State Anxiety – STAI1				
Positive (%)	11 (10.7)	4 (6.3)	7 (17.9)	0.095
Score (mean ±SD)	42.7 ± 5.1	42.1 ± 5.1	43.7 ± 5.0	0.127
Trait Anxiety – STAI2				
Positive (%)	7 (6.8)	4 (6.3)	3 (7.7)	>0.99
Score (mean ±SD)	42.1 ± 4.8	42.4 ± 4.7	41.7 ± 5.1	0.511

Note

RA: Rheumatoid Arthritis; SpA: Seronegative Spondyloarthropathies; *SD*: Standard Deviation; BDI‐II: Beck Depression Inventory II; STAI1: State‐Trait Anxiety Inventory 1; STAI2: State‐Trait Anxiety Inventory 2.

### 3.3 Sleep questionnaires

Mean total PSQI score was 6.3 ± 3.6 points (range 1–19), suggestive of poor sleep quality. 65 (63.1%) patients obtained a PSQI score ≥5, without any significant difference between RA and SpA groups.

Twenty‐five patients (24.3%) resulted at high risk for OSAS at the Berlin questionnaire. SpA patients resulted significantly more likely to be at risk than RA patients (*p* = 0.036). Category 3 of the Berlin questionnaire was significantly more positive in SpA patients. (*p* = 0.033) (Table 3).

**Table 3:** Results of sleep questionnaires further stratified by IA type

	Total (103 pts)	RA (64 pts)	SpA (39 pts)	*p*
Pittsburgh sleep quality index				
Score >5 (%)	65 (63.1)	39 (60.9)	26 (66.7)	0.525
Score (mean ±SD)	6.3 ± 3.6	6.2 ± 3.8	6.4 ± 3.2	0.797
Berlin questionnaire				
High risk of OSAS (%)	25 (24.3)	11 (17.2)	14 (35.9)	**0.036**
Score (median, 1st Q−3rd Q)	1 (0–1)	1 (0–1)	1 (0–2)	0.147^a^
Category 1 – Positive (%)	48 (46.6)	30 (46.9)	18 (46.2)	>0.999
Category 2 – Positive (%)	13 (12.6)	7 (10.9)	6 (15.4)	0.555
Category 3 – Positive (%)	36 (35.0)	17 (26.6)	19 (48.7)	**0.033**

Note

RA: Rheumatoid Arthritis, SpA: Seronegative Spondyloarthropathies, *SD*: Standard Deviation, OSAS: Obstructive Sleep Apnea Syndrome, Q: quartile.

Bold values are used to identify statistically significant results (*p*<0.05).

a The Mann‐Whitney test was used for this variable.

### 3.4 REM sleep parasomnias

Only two female patients (71 and 47 years old) fulfilled the criteria for clinical RBD. Both had a diagnosis of RA. The older patient was in an active disease phase. Her RA had begun at 54 years of age and RBD at 65 years during an active phase of the arthropathy. RBD consisted in vocalizations, head and limb movements associated with frightening dreaming scenes occurring twice a week. No changes in frequency, intensity or clinical manifestation were reported over the years. The other patient presented RA at 38 years and RBD approximately 20 years before RA onset. RBD episodes were characterized by movements associated with frightening dreams and had a fluctuating course with periods of higher (once a week) and lower frequency (3–4 per year). Periods of higher frequency were apparently not correlated with RA activity.

**Figure 1:**
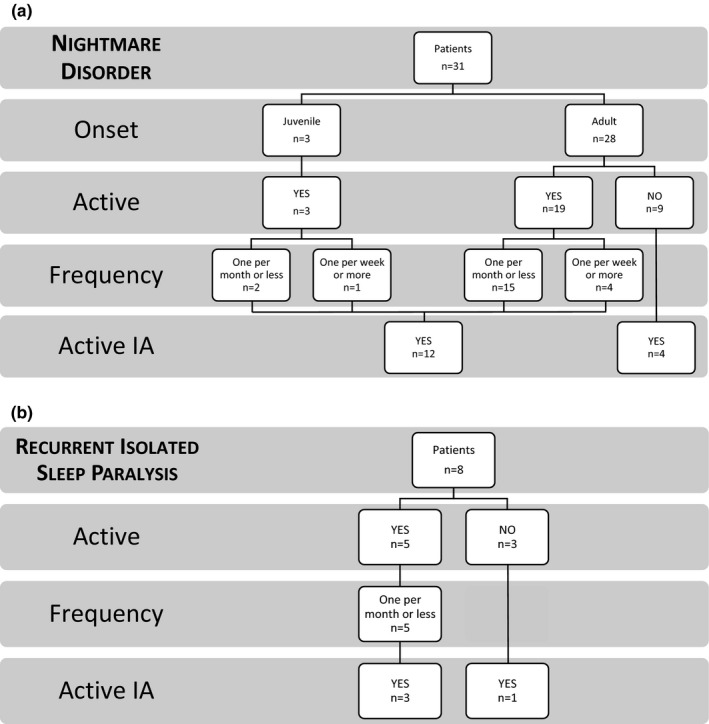
Distribution and features of REM sleep parasomnias other than RBD in our IA cohort

Thirty‐one patients (30%; 10 males) fulfilled the criteria for nightmare disorder (ND), 22 (34%) were diagnosed with RA, 9 (23%) with SpA. Nightmares started at a mean age of 31.1 ± 19.7 years; only 3 patients reported a juvenile (before 13 years old) onset. ND started 10.1 ± 13.1 years before IA onset; 4 patients referred the onset of nightmares within 5 years from the first rheumatic symptoms (Figure 1a). Twenty‐two (71%) patients reported ND as active (i.e., at least one episode in the last 6 months) and 5 (23%) of them reported one or more nightmares per week. 12 (54%) out of 22 patients were in an active phase of the arthropathy. DMARDs use was not significantly more prevalent in patients with ND (*p* = 0.71).

Eight patients (8%; two males) scored positive for recurrent isolated sleep paralysis (RISP) criteria, 6 (9%) were diagnosed with RA and 2 (5%) with SpA. Five patients recalled the age at onset of episodes at a mean age of 20.8 ± 7.2 years; in 4 of them RISP started before IA onset, in one 7 years afterwards (Figure 1b). 5 (63%) patients reported the sleep disorder as active (i.e., at least one episode in the last 6 months), three of whom presented with concurrent active IA.

Five patients (5%) scored positive for more than one REM parasomnia criterion: 4 of them reported both ND and RISP, 1 ND and RBD. Summing up, 36 (35%) patients presented positive criteria for REM sleep parasomnias.

## 4 DISCUSSION

To the best of our knowledge, this is the first study to evaluate clinical RBD in a cohort of autoimmune and inflammatory disease patients such as IA. Two (1.9%) female patients in our sample were positive for pRBD.

Previous publications described RBD prevalence in the general population (Haba‐Rubio et al., 2018; Ohayon et al., 1997) or in neurology‐driven cohorts such as sleep centers (Frauscher et al., 2010) without focusing on the possible role of an endangering condition such as IA on RBD development. An RBD cohort study found a higher prevalence of autoimmune disorders in early onset female patients, but this was a secondary analysis in a retrospective study (Ju, Larson‐Prior, & Duntley, 2011). On the other hand, papers describing sleep features in rheumatologic populations addressed exclusively sleep quality (In et al., 2016; Løppenthin et al., 2015), sometimes relating it to affective disorders (DaCosta, Zummer, & Fitzcharles, 2009) and/or sleep related respiratory problems (Drossaers‐Bakker, Hamburger, Bongartz, Dijkmans, & Soesbergen, 1998).

No study has hitherto specifically looked for RBD in IA in the light of the theories proposed by Hobson and Horowski. They hypothesized and described the anticipatory role of REM sleep and dreaming improving reaction and procedural preparation to respond to potentially dangerous events (Hobson, 2009; Horowski et al., 2004). We further speculated that disruption of physiological REM sleep regulation in RBD could represent not only the harbinger of a prominent neurodegenerative disease (Schenck et al., 2013), but could also be a red flag signaling an internal danger such as the autoimmune inflammatory activation found in IA. Therefore, our study sought clinical proof of this neurobiological hypothesis and evaluated the role of autoimmune inflammatory‐mediated diseases such as IA in the genesis of RBD. Pathophysiologically, inflammatory and autoimmune conditions connected to RBD have only been described in immunological disorders directly affecting the brain such as narcolepsy (AASM, 2014) and frequently described in case reports or series (Cornelius et al., 2011; Iranzo et al., 2006; Plazzi & Montagna, 2002). Cohort studies consist in secondary findings related to autoimmune diseases (Ju et al., 2011) or the immune typing of otherwise not inflammatory‐affected patients (Schenck, Garcia‐Rill, Segall, Noreen, & Mahowald, 1996; Schenck, Ullevig, Mahowald, Dalmau, & Posner, 1997).

Our estimated prevalence in a cohort with a hypothesized higher risk of RBD is substantially consistent with the evidence in the general population around 1%–2% (Haba‐Rubio et al., 2018). First, our result could imply that isolated RBD only manifests in neurodegenerative diseases or as a harbinger of their development (Schenck et al., 2013), weakening the relation between autoimmunity and iRBD and disproving our adaptation of Hobson's theory on REM and dreaming. The only cohort study suggesting a connection between isolated RBD and autoimmunity (Ju et al., 2011) and showing a high prevalence (20%) of autoimmune diseases among women with RBD presented a referral bias, a high rate of antidepressant usage and an inverse correlation between sleep and immunity as possible confounders. These cannot be applied to our study as we assessed a rheumatologically‐oriented cohort and we not only ruled out any concurrent use of antidepressants but also any clinical sign compatible with an ongoing neurological disease. Second, the low RBD prevalence could also be explained by cohort selection. IA is prominent among autoimmune diseases with systemic involvement (Cooper, Bynum, & Somers, 2009) and even if the primary central nervous system condition which could cause a lesional RBD is absent or extremely rare (Bougea et al., 2015), IA patients are not the only ones with these features. This could imply that the inflammation underlying these manifestations is not related to or is not sufficient to cause RBD, but that other autoimmune processes could.

Unexpectedly, we clinically confirmed active ND in 22 (21%) patients, 19 (18%) presented with adolescent‐adult onset, characterized by recurrent highly dysphoric dreams often resulting in awakening and causing clinically significant distress or impaired functioning (AASM, 2014). Our prevalence was more than 2‐fold higher than that in the general population, ranging from 2% to 8% (AASM, 2014), even excluding patients with juvenile onset. In our cohort, a typical nightmare episode was described by a 70 years old female patient dreaming an anguishing situation where she knew her nephew was in high danger, but she was unable to do anything for him in spite of her efforts. Several episodes ascribed to nightmares were characterized by more structured oneiric contents such as in a 57 years old male patient who dreamt of being threatened by feral birds of prey during one of his usual haunting trips, causing to him the distressing sensation of being backed into a corner. Finally, some patients described more vivid and complex scenes such as being attacked by a criminal during a walk with family members in the city center.

These results suggest two important considerations: (a) nightmares could represent a milder or unrecognized form of an underlying RBD, (b) nightmares and not RBD could be a sign of disrupted physiological dreaming representing a red flag signaling an internal danger underway.

ND is usually more associated with dysphoric dreams causing distress to the patient, rather than with the vivid, intense and negatively toned dreams of RBD (AASM, 2014). However, using a semi‐structured interview following the criteria of ICSD‐3 we cannot exclude that nightmares could be otherwise unrecognized RBD episodes. This is compatible with the results of a large cohort study showing that only 17.5% of RBD patients diagnosed in a sleep center were referred to the clinic for suspected RBD (Frauscher et al., 2010). Moreover, milder forms of the disorder are usually not perceived by patients, typically women (Schenck & Mahowald, 2002) who could recall just the vivid and frequently frightening dreams and who made up 65% of our cohort.

On the other hand, whether nightmares are simply homeostatic processors of emotions or symptoms of underlying clinical conditions remains a major issue in modern sleep medicine (Nielsen & Carr, 2017): they are more than simply “bad dreams”. In addition, Valli's evolutionary theory on threat simulation suggested that the purpose of dreaming (and nightmares) is to create a virtual environment to confront and to cope with threatening situations (Valli et al., 2005), similarly to Hobson's and Horowski's anticipatory role (Hobson, 2009; Horowski et al., 2004). Applied to our findings, as a chronic rheumatologic disease IA could represent the underlying condition of internal threat in our patients. Only half (12/22; 55%) of active ND patients presented with an underlying active phase of IA and just 4 (18%) reported a clear time correlation with IA symptoms onset, suggesting that chronic inflammatory activation more than its acute relapses or its onset has to be considered in ND origin. However, it has yet to be verified whether it is IA itself that triggers nightmares or whether the patient's perception of a dangerous condition is the real cause of the “bad dreams”. Indeed, depression or anxiety as important contributors to ND (Spoormaker, Schredl, & Bout, 2006) seems to be partially disproved as only 11 (35.5%) patients out of 31 with ND scored positive for depression and 4 (12.9%) presented higher levels of trait anxiety. As our work studied nightmares from the retrospective recollection of patients’ memories, only a prospective enquiry on the topic could solve the matter.

RISP was present in 8 (8%) patients and clinically active in 5 (4.9%) consistent with the prevalence of 5%–6% in young and adult (Ohayon, Zulley, Guilleminault, & Smirne, 1999) general population.

Finally, this study characterized sleep in a cohort of rheumatologic patients. Our results confirmed that poor sleep quality is common in IA patients (In et al., 2016; Løppenthin et al., 2015) often comorbid with anxiety and depression (DaCosta et al., 2009). Moreover, OSAS affected up to one fourth of IA patients in our population, concordant with literature data (Drossaers‐Bakker et al., 1998). A higher risk for OSAS is often associated with higher BMI (AASM, 2014) as shown in the SpA sub‐cohort displaying a significant positivity to the questionnaire. Indeed, patients with PA are more likely to be obese (Herron et al., 2005).

## 5 STRENGTHS AND LIMITATIONS

Our study presents several limitations. First, clinical (or probable) RBD was investigated by means of a detailed interview verifying ICSD‐3 criteria, so the RBD diagnosis was not made using the gold standard videopolysomnography (vPSG), which was not available and easily adaptable into a purely rheumatologic setting. Moreover, as only two patients were positive for RBD it was impossible to correlate RBD with IA activity and larger studies are required to disclose this correlation. Finally, the study setting precluded neuroradiological examination (namely brain MRI or CT), especially in RBD positive patients to exclude potentially lesional forms.

Equally, our study presents some strengths. It is the first to explore RBD prevalence in a specific cohort of autoimmune patients objectively evaluating neurobiological hypotheses. In addition, we used an interview with a trained neurologist rather than questionnaires to identify probable RBD patients. This not only enhanced the setting of clinical RBD diagnosis but also increased the specificity of our investigation, which is the main concern for RBD questionnaires (Bolitho et al., 2014). In fact, our estimated prevalence (1.9%) is more similar to vPSG‐based studies (1%–2%) (Haba‐Rubio et al., 2018) than questionnaire‐based investigations (4.6%–7.7%) (Mahlknecht et al., 2015).

## 6 CONCLUSIONS

This cohort study found a 1.9% prevalence of RBD in patients with rheumatologic autoimmune diseases, similar to that of the general population, whereas ND presented a 2‐fold increased prevalence. Whether RBD can be considered a red flag signaling an internal danger remains an open question, while ND may be a new player in this intriguing relation.

## CONFLICT OF INTERESTS

This was not an industry supported study. This work was performed at the Istituto Ortopedico Rizzoli, Medicine and Rheumatology Unit, DIBINEM, University of Bologna, Bologna, Italy. The study was partially supported (Dr. Provini) by a grant of the Italian Ministry of Health (“Ricerca Corrente”). L. Baldelli declares no personal financial conflicts of interest. O. Addimanda declares no personal financial conflicts of interest. M. Burattini declares no personal financial conflicts of interest. G. Chiaro declares no personal financial conflicts of interest. V. Brusi declares no personal financial conflicts of interest. E. Pignotti declares no personal financial conflicts of interest. R. Meliconi declares no personal financial conflicts of interest. F. Provini received fees for consultancy and speaking engagement from Sanofi, Fidia, Bial, Vanda Pharmaceutical, Zambon, Eisai Japan, Italfarmaco.

## AUTHOR CONTRIBUTION

L. Baldelli and O. Addimanda collaborated in data collection, performed database creation and manuscript composition. M. Burattini, G. Chiaro and V. Brusi collaborated in data collection and database organization. E. Pignotti performed and supervised data statistical analysis. F. Provini and R. Meliconi conceptualized and designed the study protocol and proofread the final version of the manuscript.
